# Clinical characteristics and genetic analysis of a case of a patient with familial hereditary breast cancer: a case report

**DOI:** 10.1186/s13256-024-04685-y

**Published:** 2024-08-14

**Authors:** Yuan Liu, Jinglin Mao, Longquan Xiang, Xiangyu Zhang, Zhen Qu

**Affiliations:** 1 Department of Surgical Oncology, First Affiliated Hospital, Bengbu Medical College, Bengbu, Anhui China; 2 Department of Pathology, Jining No. 1 People’s Hospital, Jining, 272000 Shandong China; 3 Department of Hepatobiliary and Pancreas, The Affiliated Hospital of Qingdao University, Qingdao University, Qingdao, Shandong China

**Keywords:** Hereditary triple negative breast cancer, Whole-exome sequencing, *MT1E*

## Abstract

**Background:**

Breast cancer has emerged as the foremost cause of female mortality worldwide, with triple negative breast cancer accounting for approximately 10–15% of all breast cancer cases. The triple negative breast cancer family has obvious familial heritability, but no potential pathogenic variation was found in *BRCA1/2*.

**Case presentation:**

The patient was a 56-year-old woman of Han ethnicity. The clinical characteristics of this patient with breast cancer were summarized, peripheral blood of one normal female and two patients with breast cancer in this family was collected, DNA was extracted, and the potential pathogenic variation was analyzed by whole exome sequencing. The normal female and two patients with breast cancer in this family shared a maternal grandmother. The proband’s right breast mass was punctured, and the biopsy showed invasive carcinoma of the right breast, grade II–III, with necrosis. No mutation was found in *BRCA1/2* gene test; immunohistochemical of surgical specimens showed triple negative breast cancer. Three mutation types and 17 gene mutation sites were detected through bioinformatics prediction analysis on the basis of co-segregation of genotype and phenotype within the family and whole exome sequencing results. Combined with the Cancer Genome Atlas database comprehensive analysis, the *MT1E* c.G107A (p.C36Y) mutation may be a potential pathogenic site.

**Conclusions:**

Through whole exome sequencing, we identified a total of 17 potential pathogenic mutation loci, none of which have been reported thus far. Therefore, our work expanded the gene mutation spectrum of familial hereditary triple negative breast cancer, which can provide more basis for family genetic counseling.

**Supplementary Information:**

The online version contains supplementary material available at 10.1186/s13256-024-04685-y.

## Background

Breast cancer has emerged as the foremost cause of female mortality worldwide, with the highest incidence and mortality rates among malignancies affecting women [1]. At present, approximately 5–10% of all patients with breast cancer are caused by germline mutations, and these patients generally show obvious familial heritability. More than 20 breast cancer susceptibility genes have been reported, including *BRCA1*, *BRCA2*, *ATM*, *RAD51*, *CHEK2*, and *PALB2*, which are involved in DNA repair. Triple negative breast cancer (TNBC) has its name due to its negative expression of estrogen receptor (ER), progesterone receptor (PR), and human epidermal growth factor receptor 2 (HER2). TNBC accounts for approximately 10–15% of all breast cancer cases and exhibits a poor prognosis, with significantly lower 5-year survival rates compared with other breast cancer subtypes. Pathogenic variants involved in the homologous recombination repair (HRR) pathway, especially *BRCA1/2*, are predominantly implicated in hereditary TNBC cases. Patients carrying *BRCA1/2* pathogenic variants can benefit from poly(ADP-ribose) polymerase inhibitors (PARPi) treatment. We collected a case of familial triple negative breast cancer without *BRCA1/2* pathogenic variation. Whole exome sequencing (WES) showed that there were some potential pathogenic variation sites in this family, which could expand the spectrum of mutations in familial TNBC.

## Case presentation

The proband was a 56-year-old Chinese Han female patient who presented to the First Affiliated Hospital of Bengbu Medical College in March 2020 with a breast mass. Clinical palpation revealed a poorly defined, immobile, hard mass measuring approximately 2 × 2 cm in the outer quadrant of the right breast. There was no tenderness upon compression, and no discharge or abnormalities were observed in the bilateral breasts or nipples.

**Fig. 1:**
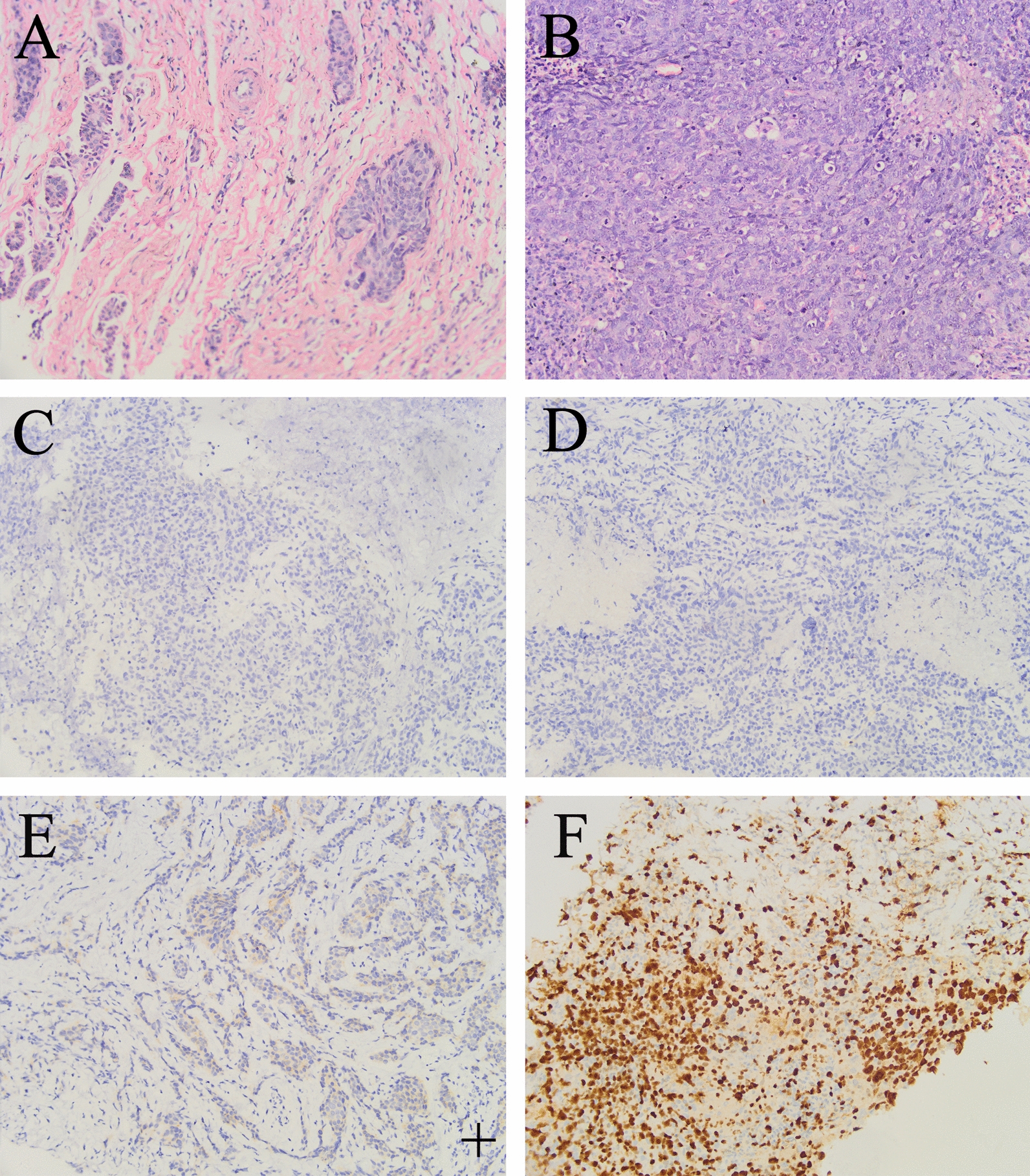
Pathological diagnosis results of the proband. **A** Pathological puncture results of the right breast of the proband shows invasive cancer. **B** Proband’s postoperative pathological diagnosis is invasive carcinoma of the right breast. **C** Estrogen receptor is negative. **D** Progesterone receptor is negative. **E** Human epidermal growth factor receptor 2 is one “+”. **F** Ki67 is positive, approximately 80%. Original magnification, 200×

Preoperative examinations, including chest computed tomography (CT) scan and electrocardiogram, showed no significant abnormalities. Color Doppler ultrasound revealed a hypoechoic lesion measuring 17 × 19 × 21 mm in the 9 o’clock position of the right breast. The lesion appeared irregular in shape with indistinct borders and exhibited lobulated margins. No apparent abnormal lymph nodes were detected in the right axilla. Molybdenum target imaging confirmed the presence of a nodule in the outer quadrant of the right breast, classified as BI-RADS 4c. Fine-needle aspiration biopsy of the right breast mass revealed invasive carcinoma, grade II–III, with necrosis (Fig. 1A). BRCA1/2 genetic testing did not identify any pathogenic variants.

On 3 April 2020, the patient underwent breast-conserving surgery + sentinel lymph node biopsy (SLNB) under general anesthesia. Intraoperative frozen section analysis of the margins (upper, lower, inner, outer, and basal) was negative. Postoperative pathology confirmed invasive carcinoma of the right breast (Fig. 1B), grade 3, measuring 2.5 × 2.3 × 1.5 cm. Immunohistochemistry analysis revealed the following results: estrogen receptor (ER) negative, progesterone receptor (PR) negative, human epidermal growth factor receptor 2 (HER2) 1+, and high Ki-67 proliferation index (+, 80%) (Fig. 1C–F).

**Fig. 2:**
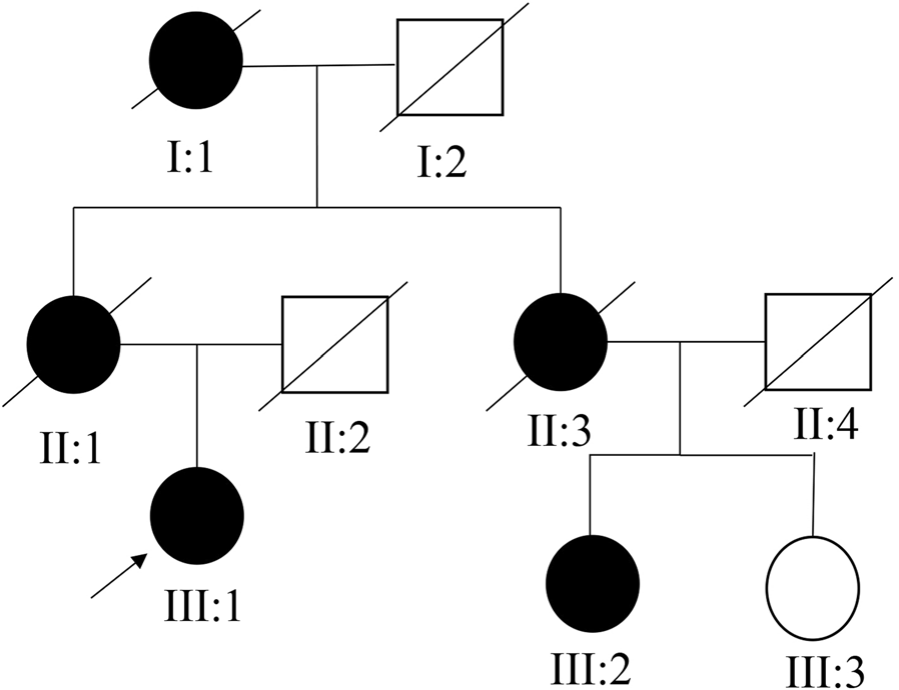
Pedigree structure of the patients with breast cancer. The circle represents female, the square represents male, black represents patient, white represents normal person, “fullwidth solidus” represents deceased, and the proband (III:1) is indicated with an arrow

According to the patient’s account, there is a clear familial predisposition to breast cancer. Therefore, information regarding the patient’s pedigree was collected (Fig. 2). As depicted in Fig. 2, all female individuals within the family, with the exception of III:3, were affected by breast cancer. Unfortunately, detailed information regarding I:1, II:1, and II:3 is not available due to their demise. Both III:2 and the proband III:1 were diagnosed with TNBC. As indicated by the family pedigree, the inheritance pattern of this disease is dominant.

According to the predetermined criteria, WES-identified a total of 17 candidate variants (Table 1), including frameshift mutations, missense mutations, and splice site mutations. In addition, WES showed that the TNBC susceptibility genes *BRCA2* and *ATM* carry likely benign variations (Supplementary Material, Table S2). Metallothionein 1E (*MT1E*) harbored a c.G107A (p.C36Y) mutation. This mutation has been found to be absent or extremely rare in databases such as 1000 Genomes, ESP6500si_all, and gnomAD_ALL (Table 2). Furthermore, on the basis of the bioinformatics predictions from dbscSNV and Spidex analysis software (Table 2), it is suggested that this mutation does not affect the splicing of *MT1E*. Multiple online prediction software tools, including SIFT, PolyPhen-2, MutationTaster, and CADD, indicate that *MT1E* c.G107A (p.C36Y) is a pathogenic variant.

**Table 1:** Summary of deleterious mutations from whole exome sequencing

Gene	Mutation type	Transcript	Site	AA change	Pathogenicity prediction
*TOP1MT*	Frameshift	NM_052963	c.765delC	p.S255Rfs*9	Deleterious
*LAMC2*	Missense	NM_005562	c.T3142C	p.S1048P	Deleterious
*RPS6KC1*	Missense	NM_001349663	c.A1483C	p.S495R	Deleterious
*SLCO2A1*	Missense	NM_005630	c.G1136A	p.G379E	Deleterious
*LSG1*	Missense	NM_018385	c.C1039T	p.R347W	Deleterious
*FYB*	Missense	NM_001465	c.A995G	p.K332R	Deleterious
*BEND3*	Missense	NM_001080450	c.C2041T	p.R681W	Deleterious
*AUTS2*	Missense	NM_015570	c.G2461A	p.A821T	Deleterious
*ZCWPW1*	Missense	NM_017984	c.C1064T	p.P355L	Deleterious
*CEL*	Missense	NM_001807	c.T2068G	p.S690A	Deleterious
*NCAPD2*	Missense	NM_014865	c.C2548T	p.R850W	Deleterious
*IGDCC3*	Missense	NM_004884	c.C2435A	p.S812X	Deleterious
*CALML4*	Missense	NM_033429	c.G442A	p.D148N	Deleterious
*MT1E*	Missense	NM_175617	c.G107A	p.C36Y	Deleterious
*PODNL1*	Missense	NM_024825	c.T1016G	p.L339R	Deleterious
*GFY*	Missense	NM_001195256	c.C67T	p.P23S	Deleterious
*LYL1*	Splice site	NM_005583	c.427+5G>A	unknown	Deleterious

**Table 2:** Pathogenicity prediction of *MT1E* c.G107A (p.C36Y) mutation

Number	Analysis tool	Mutation rate	Risk prediction	Score
1	1000g_ALL	0	–	–
2	esp6500si_all	0	–	–
3	GnomAD_ALL_AF	0.00004690	–	–
4	dbscSNV_SCORE	–	–	None
5	Spidex	–	–	None
6	SIFT	–	Deleterious	0
7	Polyphen2_HVAR	–	Deleterious	0.998
8	Polyphen2_HDIV	–	Deleterious	0.997
9	MutationTaster	–	Deleterious	1
10	CADD	–	Deleterious	27.3

**Fig. 3:**
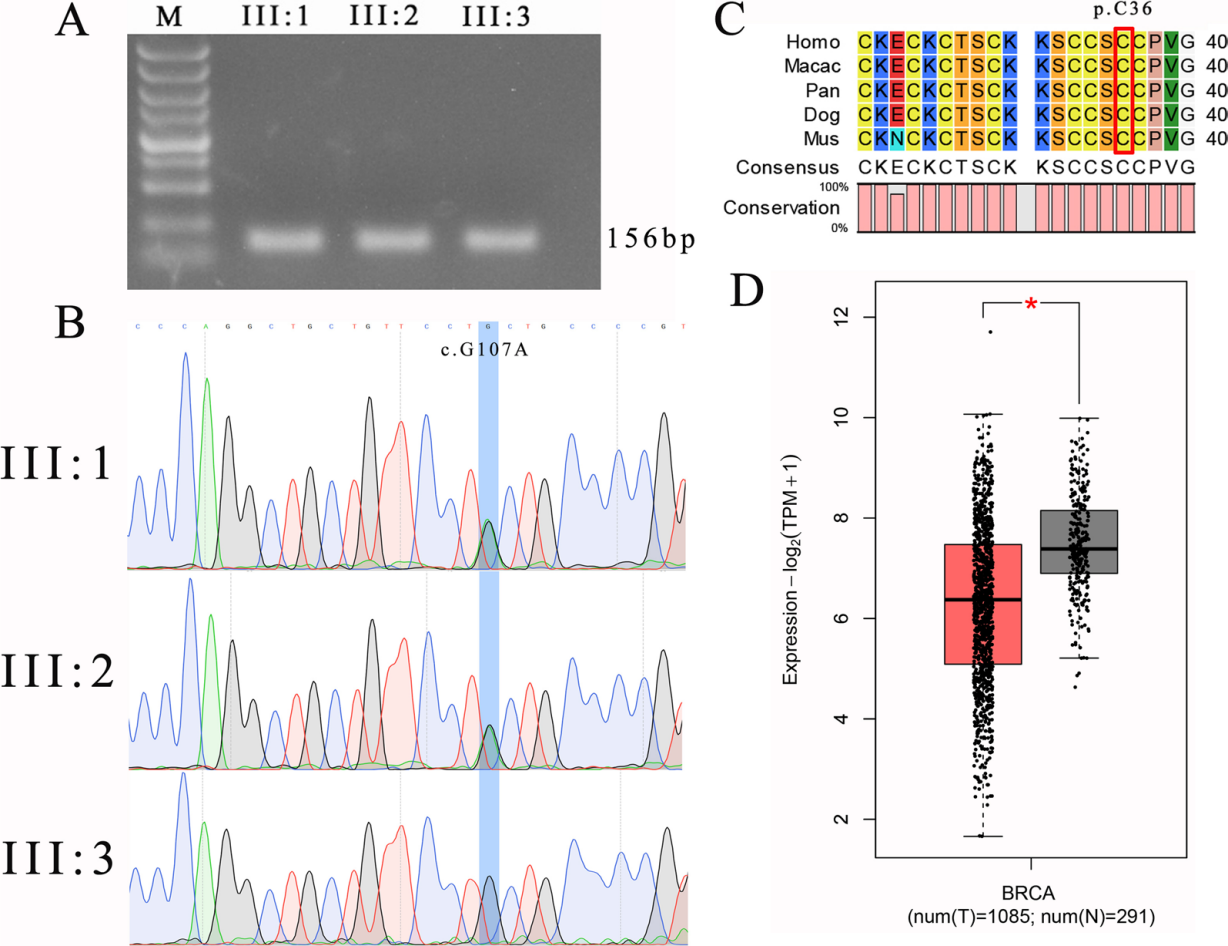
Conservative and expression analysis of Metallothionein 1E. **A** Agarose gel electrophoresis of polymerase chain reaction amplification fragment. Polymerase chain reaction fragment is 156 bp and contains the Metallothionein 1E c.G107A mutation site. **B** Sanger sequencing confirms the mutation. Polymerase chain reaction fragments are sequenced by Sanger, and the mutation is consistent with whole exome sequencing sequencing. **C** Metallothionein 1E p.C36 is conserved in different species such as human, macaque, chimpanzee, dog, and mice. **D** Analysis of Metallothionein 1E expression in breast cancer and para-cancer tissues in the Cancer Genome Atlas database. Red represents breast cancer, gray represents normal tissue; *BRCA* breast cancer

Initially, we amplified a 156 bp polymerase chain reaction (PCR) fragment (Supplementary Material, Table S1) containing the *MT1E* c.G107A mutation (Fig. 3A, Supplementary Material, Fig. S1) and performed Sanger sequencing. The results confirmed the presence of the c.G107A mutation in both III:1 and III:2, while III:3 did not carry this mutation (Fig. 3B), consistent with the WES results. Furthermore, we used CLC Sequence Viewer8 software to analyze the conservation of the cysteine residue at position 36 of the MT1E protein across different species, including humans, macaques, chimpanzees, dogs, and mice. The results indicated a high level of conservation for p.C36 among these species (Fig. 3C).

To investigate the expression of* MT1E* in breast cancer compared with normal tissues, we utilized an online bioinformatics analysis system (GEPIA2, http://gepia2.cancer-pku.cn/#general) to analyze the expression of *MT1E* in cancer and para-cancer tissues in the the Cancer Genome Atlas (TCGA) database. The analysis revealed a significant downregulation of *MT1E* in breast cancer tissues (Fig. 3D).

The conservation analysis demonstrated the high conservation of MT1E p.C36 across different species. Bioinformatics analysis indicated that the *MT1E* c.G107A mutation (p.C36Y) has an extremely low frequency and is highly pathogenic in the population. Additionally, compared with breast cancer tissues, the expression of *MT1E* is higher in the para-cancer tissues, which indicates that *MT1E* may play an important role as a tumor suppressor gene. Further, the *MT1E* c.G107A mutation may downregulate the expression of *MT1E*, potentially contributing to the development of breast cancer.

## Discussion and conclusions

On the basis of the pedigree analysis, we could confirm the familial inheritance of TNBC as dominant but could not further determine whether it follows an autosomal or X-linked pattern [2]. Additionally, due to the majority of family members being deceased, further investigation of potential pathogenic genes and variant sites through co-segregation analysis was not feasible.

Through WES analysis of two patients and one normal individual within the pedigree, we filtered the mutation sites and performed bioinformatics analysis, resulting in a total of 17 variant sites. Our study provides evidence that the *MT1E* c.G107A (p.C36Y) mutation has a very low frequency in the general population and is predicted to be pathogenic by multiple bioinformatics tools. Moreover, the high conservation of MT1E throughout evolution and the conservation of the p.C36 residue across species further support the potential significance of this mutation in relation to breast cancer. However, further functional studies are needed to gain a deeper understanding of the molecular mechanisms underlying the role of this mutation in breast cancer pathogenesis.

MT1E is a member of the metallothionein family, which generally exhibits high affinity for heavy metal ions such as zinc and mercury [3]. Previous studies have shown that in human glioma cell lines, MT1T can promote tumor cell migration and invasion through the regulation of the MT1E-NF-κB p50-MMP-9 signaling pathway [4]. However, in studies on prostate cancer and hepatocellular carcinoma, MT1E has been found to act as a tumor suppressor gene, promoting cancer cell apoptosis and inhibiting cell proliferation and metastasis [5, 6]. In breast cancer research, elevated methylation levels of MT1E have been observed in patients with triple negative breast cancer with positive KI-67 expression [7]. Higher methylation levels of MT1E indicate lower gene expression levels, which is consistent with our experimental results. Currently, research on MT1E remains limited, and its specific molecular mechanisms have yet to be elucidated.

In summary, our study suggests that the *MT1E* c.G107A (p.C36Y) variant may be an important mutation associated with the development of TNBC. In addition, this work may expand the spectrum of mutations in familial TNBC, and also provide more clues for subsequent triple negative breast cancer research.

### Supplementary Information

Supplementary Material 1.

## Data Availability

The datasets used and/or analyzed during the current study are available from the corresponding author on reasonable request.

## Abbreviations

MT1E: Metallothionein 1E
TNBC: Triple negative breast cancer
WES: Whole-exome sequencing
ER: Estrogen receptor
PR: Progesterone receptor
HER2: Human epidermal growth factor receptor 2
HRR: Homologous recombination repair
