# Supplementary material for: Clinical characteristics and genetic analysis of a case of a patient with familial hereditary breast cancer: a case report
Source: J Med Case Rep. 2024 Aug 14;18:368. doi: 10.1186/s13256-024-04685-y (PMC11323601; doi:10.1186/s13256-024-04685-y)
Supplement: Supplementary file 1 — Supplementary Material 1. [file 13256_2024_4685_MOESM1_ESM.docx]

Supplementary Material



Figure S1:

Figure S1: Original image of agarose electrophoresis gel. Sample loading twice.

Table S1:

Table S1: Amplification primer sequence

| Primer name | Primer sequence |
| --- | --- |
| MT1E-forward primer  MT1E-reverse primer | CTCGAGCCAGGCTTGCTATT  CACTTCTCCGATGCCCCTTT |

Table S2：Summarize the most frequent TNBC genes mutation in this family

| Gene | Is this family involved in | Variant site | SNP ID | Pathogenicity prediction |
| --- | --- | --- | --- | --- |
| BRCA1  BRCA2  PALB2  CHEK2  ATM  CDH1  TP53  STK11  PTEN | No  Yes  No  No  Yes  No  No  No  No | -  c.G8187T:p.K2729N  -  -  c.A125G:p.H42R  -  -  -  - | -  rs80359065  -  -  rs201773026  -  -  -  - | -  Likely-benign  -  -  Likely benign  -  -  -  - |

Note: TNBC: Triple negative breast cancer
